# Characteristics of TCR Repertoire Associated With Successful Immune Checkpoint Therapy Responses

**DOI:** 10.3389/fimmu.2020.587014

**Published:** 2020-10-14

**Authors:** Joel Kidman, Nicola Principe, Mark Watson, Timo Lassmann, Robert A. Holt, Anna K. Nowak, Willem Joost Lesterhuis, Richard A. Lake, Jonathan Chee

**Affiliations:** ^1^National Centre for Asbestos Related Diseases, Institute of Respiratory Health, University of Western Australia, Perth, WA, Australia; ^2^School of Biomedical Sciences, University of Western Australia, Perth, WA, Australia; ^3^Institute for Immunology and Infectious Diseases, Murdoch University, Perth, WA, Australia; ^4^Telethon Kids Institute, Perth, WA, Australia; ^5^Canada’s Michael Smith Genome Sciences Centre, Vancouver, BC, Canada; ^6^School of Medicine, University of Western Australia, Perth, WA, Australia

**Keywords:** checkpoint immunotherapy, tumor immunology, T cell receptor, immunogenomics, dynamic analysis

## Abstract

Immunotherapies have revolutionized cancer treatment. In particular, immune checkpoint therapy (ICT) leads to durable responses in some patients with some cancers. However, the majority of treated patients do not respond. Understanding immune mechanisms that underlie responsiveness to ICT will help identify predictive biomarkers of response and develop treatments to convert non-responding patients to responding ones. ICT primarily acts at the level of adaptive immunity. The specificity of adaptive immune cells, such as T and B cells, is determined by antigen-specific receptors. T cell repertoires can be comprehensively profiled by high-throughput sequencing at the bulk and single-cell level. T cell receptor (TCR) sequencing allows for sensitive tracking of dynamic changes in antigen-specific T cells at the clonal level, giving unprecedented insight into the mechanisms by which ICT alters T cell responses. Here, we review how the repertoire influences response to ICT and conversely how ICT affects repertoire diversity. We will also explore how changes to the repertoire in different anatomical locations can better correlate and perhaps predict treatment outcome. We discuss the advantages and limitations of current metrics used to characterize and represent TCR repertoire diversity. Discovery of predictive biomarkers could lie in novel analysis approaches, such as network analysis of amino acids similarities between TCR sequences. Single-cell sequencing is a breakthrough technology that can link phenotype with specificity, identifying T cell clones that are crucial for successful ICT. The field of immuno-sequencing is rapidly developing and cross-disciplinary efforts are required to maximize the analysis, application, and validation of sequencing data. Unravelling the dynamic behavior of the TCR repertoire during ICT will be highly valuable for tracking and understanding anti-tumor immunity, biomarker discovery, and ultimately for the development of novel strategies to improve patient outcomes.

## Introduction

Immunotherapies that harness T cell responses against cancer have changed cancer treatment. Therapies such as immune checkpoint therapy (ICT) and adoptive T cell transfer now play a critical role in the treatment of solid and blood malignancies. In-depth understanding of the biology that underlies immunotherapy success or failure is crucial for treatment monitoring and improving current therapies. Cutting edge high-throughput sequencing and flow cytometry have enabled multi-faceted profiling of T cells, evaluating immune receptor composition, antigen specificity, epigenetic and functional status of T cells, greatly contributing to our understanding of how the anti-tumor T cell responds especially in the context of ICT.

## ICT is a Revolutionary Cancer Therapy, but Not All Patients Respond

Treatment with antibodies that block inhibitory receptors, such as cytotoxic T lymphocyte associated protein 4 (CTLA-4), programmed death receptor 1 (PD-1), or its ligand PD-L1, can lead to durable complete responses in some patients depending on the cancer type (1). CTLA-4 blockade has been the most successful in metastatic melanoma, while responses in other cancers such as non-small cell lung (NSCLC) (2, 3), Hodgkin’s lymphoma (4), Merkel-cell carcinoma (5), triple-negative breast cancer (6), renal cell carcinoma (7), urothelial bladder (8, 9) and squamous cell carcinoma of the head and neck (10) are common with anti-PD-1/PD-L1 therapy.

Despite these promising results, it is difficult to predict whether an individual will benefit from ICT or not. ICT removes T cell suppression indiscriminately, causing immune related adverse events in up to 90% of treated patients, with serious autoimmune-like toxicity observed in approximately 2–5% of treated patients (11). Immune related adverse events are observed with either anti-CTLA-4 or anti-PD1/L1 therapy and increase in incidence with combination therapy. ICT is also expensive, costing approximately USD6,000 to 20,000 per patient each month, depending on the cancer type and treatment schedule (12). Importantly, only a minority of patients respond to ICT, highlighting a need to develop accurate biomarkers of response. The most clinically advanced, pre-treatment biomarkers of ICT responses include CD8^+^ T cell tumor infiltration (13, 14), intra-tumoral PD-L1 expression (13, 15), tumor mutation burden and neo-antigen burden (16, 17). However, these have poor positive and negative predictive value as pre-treatment biomarkers of ICT response and are not reproducible across all cancers.

## Measurements of Dynamic Change in the Immune System Could Offer a Biomarker of ICT Response

Although a pre-treatment predictor of ICT response would be ideal, an early on-treatment biomarker could also have value. We previously argued the therapeutic response to ICT can be visualized as a critical state transition of a complex system because of its dichotomous nature; some patients experience rapid tumor regression, but other patients do not benefit from ICT at all (18). In such complex, highly connected systems, not all determinants of response can be found in pre-treatment. Small differences in the initial state (*e.g.* minor differences in T cell repertoire) can be easily amplified in cascading events, resulting in a dramatic shift in the system state. Biomarkers could be identified between the start of treatment and when the critical state transition occurs. In the context of ICT, dynamic changes in features of the immune system, such as the T cell repertoire shortly after initiation of treatment, could inform ICT responses and biomarker development. We envisage that a dynamic biomarker will complement existing ones and facilitate clinical decisions once treatment has started. For example, dynamic biomarkers would allow the identification of patients with ‘pseudoprogression’ (an initial increase in tumor diameter due to immune cell infiltration and edema, followed by regression) who would benefit from continuing therapy, and it would identify early-on patients who will not benefit, thus limiting side effects and reducing the substantial costs associated with continued treatment. Characterizing the T cell repertoire is useful for developing potential dynamic biomarkers of ICT response, and there are different technologies used to approach this.

## Sequencing Technology Is Important for the Characterization of TCR Repertoires

**Figure 1:**
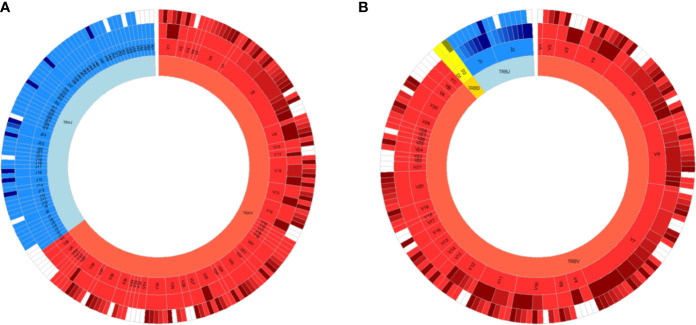
All germline VDJ gene segments of the human T cell receptor. Variable (V) genes in red, diversity (D) genes in yellow and joining (J) genes in blue. From outmost ring to innermost ring are the V (D)J sub-group, V (D)J group, and alpha **(A)**/beta **(B)** chains. Non-functional alleles are in white.

Fine characterization of T cell repertoires is made possible by the application of high-throughput sequencing. Immune specificity is derived from T cell receptors (TCRs) expressed on the surface of all T cells that bind to peptides in the context of major histocompatibility complex (MHC) proteins. Conventional T cells express a vast range of TCRs, and each TCR is typically composed of a heterodimer of *α* and *β* chains. This diversity is generated during random, somatic rearrangement of variable (V), joining (J), and diversity (D) gene segments in TCR chains (19). Most TCR diversity arises from the *β* chain because it utilizes an additional D segment (**Figure 1**). Furthermore, the process of gene rearrangement adds and removes random nucleotides between segments (20), forming a hyper-variable third complementarity-determining region (CDR3) that is a key component of specificity. Although an upper bound of 10^16^ possible unique TCR*αβ* pairs can occur, 10^4^ unique TCR*β*s can be typically routinely sampled in human peripheral blood samples (21, 22). In this review, we refer to TCR repertoire as the collection of TCRs within a given T cell population.

Sequencing across the CDR3 region of TRBV gene in either bulk populations of cells or at the single-cell level can be used to fingerprint a repertoire (21, 23, 24). There are several well-established TCR sequencing protocols, which involve targeted PCRs to generate amplicons across the CDR3 region. We briefly review commonly used protocols, as in-depth assessment and head-to-head comparisons of different techniques have been reviewed elsewhere (25–27).

TCR sequencing libraries can be prepared from genomic DNA (gDNA) or messenger RNA (mRNA) starting material, both with pros and cons (28). Essentially, DNA is more stable and can be isolated from frozen or FFPE samples, whereas there are more TCR RNA transcripts compared to the single copy of rearranged DNA. Multiple fixed forward and reverse primers specific to TCR V*β*, and TCR J*β* gene segments respectively are used in multiplex PCRs to generate TCR libraries (24). Because there are multiple TCR V*β* and TCR J*β* gene combinations, multiplex PCR is required to cover most variations (24). The benefit of the DNA approach is the direct quantification of single TCR clones as each T cell contains one TCR*β* gene rearrangement. However, transcriptional information is only contained in RNA. Both RNA and gDNA based TCRseq have well documented methods and choosing one is dependent on individual use cases.

5′ rapid amplification of cDNA ends (5′RACE) with template switching oligonucleotides is the most widely used RNA based approach to generate TCR sequencing libraries (29). This approach incorporates an adaptor site at the 5′ end of a TCR template during first strand synthesis, paired with a 3′ primer specific for the TCR constant region. Subsequent PCR amplification is performed with primers specific for 3′ end and the 5′ adaptor sequence. 5′RACE is widely used because it circumvents the need for multiple fixed primers. RNA approaches measure expressed TCR*β*s, but quantification of clonal expansion is challenging when sequencing bulk populations of T cells, because multiple copies of the same TCR could be expressed within a single cell (30). In a recent study, matched samples underwent single-cell and bulk TCR sequencing, and the proportion of each unique TCR*β* sequence highly correlated between the two approaches (31). This suggests that TCR transcript counts detected by bulk TCRseq can be representative of T cell clonal expansion, rather than increased expression of TCRs in a limited number of cells.

Regardless of starting material, PCR-based approaches are often susceptible to amplification bias, which distort the relative abundances of the sequenced products. A challenge lies in distinguishing genuine rare clones from sequencing errors, especially if sequences differ by a few nucleotides. Errors can be corrected during analysis. For example, low frequency TCR clones are often clustered with highly similar clones with significantly greater frequencies because it is more likely the sequence differences arising from these clones are due to error. We also highlight the utility of unique molecular identifiers (UMIs) or adjusting primer concentrations to correct this error (32–34). UMIs are strings of random nucleotides added between the adaptor sequence and oligonucleotides of the template switching primer. During first-strand synthesis, each cDNA template is tagged with a UMI which is carried through the entire PCR and sequencing process. Post sequencing analysis identifies sequences that originate from the same starting priming molecule based on UMI sequence, allowing for correction of any PCR bias and sequencing errors. Longer UMI sequences lower the chance of identical but distinct RNA molecules binding the same UMI (35). UMIs of 9 to 12 nucleotides are used in most RNA based TCR sequencing protocols because it provides coverage for a typical range of 10^5^ to 10^8^ distinct TCR transcripts. Repeated deep sequencing of human peripheral blood T cells identified a lower bound of 10^6^ unique TCR*β* chains in a repertoire (36). With 12 nucleotide UMIs there is sufficient coverage to sequence each unique human peripheral blood TCR*β* at most 100 times. Both DNA and RNA based bulk TCRseq approaches are now available through commercial services or kits. While it is generally accepted that current TCR sequencing methods will not capture the entirety of repertoire diversity in an individual (36), there is significant overlap between different bulk TCR sequencing approaches in capturing the most abundant and frequent TCRs.

Single-cell analysis is at the forefront of TCR sequencing technology. In addition to sequencing TCRs, single-cell sequencing interrogates transcriptional activity of individual cells, linking T cell phenotype and specificity (37). With single-cell technology, each cDNA molecule is barcoded to a unique cell in a microfluidic droplet. UMIs are added and amplicon libraries are similarly generated for sequencing. Sequencing reads from individual cells are identified through their unique barcodes. Single-cell fluidic platforms such as 10× Chromium capture transcript and TCR information from up to 10,000 of individual cells in parallel. Bulk TCRseq captures 10^5^ transcripts that provides more opportunity to sample low expanded T cell clones. Although bulk TCRseq does not provide transcriptomic information, it gives a more accurate estimation of diversity than single-cell sequencing, and is considerably cheaper. Both single-cell and bulk TCR sequencing approaches have been used in combination because they offer complementary information (reviewed in later sections). The accessibility of the assays has led to an increase in its use in immuno-oncology studies to characterize T cell repertoires, especially in the context of response/non-response to ICT.

## TCR Repertoires Can be Characterized by Different Metrics

It is important to understand how multiple TCR clones are distributed in T cell repertoires of patients that respond to ICT. As TCRseq data sets typically contain millions of TCR sequences, with unique TCR clonotypes expressed at variable frequencies, diversity metrics adapted from ecological studies are used to characterize the relative distributions of multiple TCR clones. These metrics are one-dimensional scores that estimate the distribution of species in any given system. In the case of TCR repertoires, each unique TCR clone represents a unique species, and the abundance of each clone represents the number of members of that species. The fraction of every unique species (unique TCR clone) over the total number of T cells (total TCRs) in the repertoire is calculated, weighted, summed and normalized to produce a summary statistic. The most commonly used metrics to characterize repertoire diversity in published studies are based on Shannon’s and Simpson’s diversity indices (38, 39). These scores range between maximum clonality (with one clone occupying the whole repertoire) and maximum evenness (with all clones occurring equally). The degree of T cell clonal expansion is estimated with these values. Importantly, diversity scores allow for statistical comparisons between different TCR repertoires within (for example before and after treatment) and between patients. Perturbations in TCR repertoire, such as changes in TCR diversity reflect immunological processes that can be analyzed to understand antigen-specific T cell responses. ICT-induced clonal expansion of antigen-specific T cells results in a reduction of TCR diversity. Conversely, increased migration of T cells into the tumor due to ICT could be reflected in an increase of TCR diversity. The diversity index also offers a possible stratification score for developing a biomarker of responsiveness to therapy, but this requires further validation. Diversity indices are one of few widely used metrics to characterize TCR repertoires in ICT (40).

Another common metric used to characterize similarities or differences in TCR repertoires is the Morisita–Horn index (41). It accounts for both the number and abundance of shared TCRs between two repertoires, and its score ranges between zero (no overlap) and one (all clones overlapping at similar frequencies). An advantage of performing TCR sequencing on serial samples is the ability to track dynamic changes in TCR clonotypes over time. For time course data, not only frequencies of individual TCR clonotypes are measured, but overlap metrics have been used as well (42). For example, an increased Morisita–Horn Index between two sequential samples could reflect the persistence and expansion of TCR clonotypes over time (43). Changes in TCR overlap and diversity over time are important dynamic measurements that could be informative of ICT outcomes, and have been assessed in different studies (44–47).

## Anti-CTLA-4 Monotherapy Remodels the Blood TCR Repertoire

Studies investigating TCR repertoire and ICT are difficult to compare because they differ in the type of cancer, type of sample (blood or tumor biopsy), and timing (pre-treatment or post-treatment) of sample collected, genetic material used for sequencing (gDNA or RNA), and different diversity metrics used for analysis. Our next section will focus on anti-CTLA-4 and anti-PD-1/PD-L1 separately, as they are the most commonly used antibodies in the clinic and have different mechanisms of action (48, 49).

CTLA-4 blockade appears to broaden blood CD8^+^ T cell responses against tumor associated antigens, increasing the number of tumor-specificities measured by peptide-MHC multimer staining when pre- and post-blood samples were compared (50). TCR*β* sequencing performed in separate studies support this, as anti-CTLA-4 increases the total number of unique TCR clones and increases TCR diversity in the blood (51). Although significant changes in TCR diversity were not observed in all studies (43, 52), an important observation with anti-CTLA-4 monotherapy is that highly different repertoires were observed pre- and post-treatment, as measured by low overlap indices (43, 48). This suggests that anti-CTLA-4 treatment drives a rapid influx of new T cell clones and broadens the circulating TCR repertoire, with minimal clonal expansion of T cells within the blood (51). The broadening of the T cell repertoire is indiscriminate in its specificity, as increased blood TCR diversity has been linked to immune-related side effects (48, 53).

Patients with a high pre-treatment blood TCR diversity score experienced more clinical benefit and increased survival with anti-CTLA-4 monotherapy in some studies (43, 54), but not others (48, 55, 56). TCR diversity scores from pre-treatment blood samples are highly variable because the peripheral blood contains the most heterogeneous populations of T cells. Although anti-CTLA-4 reshapes the blood TCR repertoire, some TCR clonotypes are still found before and after treatment. The persistence and expansion of high-frequency TCR clonotypes post-treatment correlate with survival in some studies (43, 51). There is no clear consensus on how TCR diversity in whole blood samples correlates with anti-CTLA-4 response.

## Anti-CTLA-4 Drives Clonal Expansion of Tumor Infiltrating Lymphocytes

There is limited clinical data about dynamic changes in tumor TCR repertoire diversity upon anti-CTLA-4 monotherapy. A reduction in overall TCR diversity (measured by Shannon’s Diversity) post-treatment compared to pre-treatment was reported in a melanoma study (57) but not in a breast cancer study (52). When TCR clones were tracked, anti-CTLA-4 drove polyclonal, rather than oligoclonal expansion of TCR clones within the tumor (52). Recent studies highlight that bystander T cells specific for non-tumor antigens can infiltrate tumors, obscuring the ability to analyze the anti-tumor T cell response (58). A significant challenge lies in distinguishing tumor-specific TCRs from the bystanders. Furthermore, most early clinical studies were performed with limited samples, and the effects of anti-CTLA-4 monotherapy are not clear because anti-CTLA-4 is mostly administered in combination with anti-PD-1/L1 or other adjuvant therapies in clinical studies. Furthermore, serial tumor biopsies are limited for most cancers. Hence preclinical models have been widely used to study the effects of anti-CTLA-4 on tumor TCR repertoires.

In murine studies, the effects of anti-CTLA-4 monotherapy on tumor TCR diversity are model-dependent. Treatment of breast tumors (4T1, E0771) results in reduced tumor TCR diversity compared to untreated tumors, and this reduction in diversity is accompanied by expansion of dominant TIL clones (59, 60). However, similar changes are not observed in murine melanoma (61, 62). B16 tumors did not respond to anti-CTLA-4 monotherapy in these studies, likely contributing to the discrepancy. *In vivo* anti-CTLA-4 treatment increases the frequency of neo-antigen specific tumor-infiltrating T cells in other responsive murine cancer lines, suggesting that clonal expansion of T cells, and reduction in tumor TCR diversity is a feature that accompanies anti-CTLA-4 treatment (63, 64). In most preclinical studies, tumors from treated vs untreated animals are compared, unlike clinical studies where serial samples from the same individual can be studied. This has to be taken into consideration when studying dynamic changes in murine models, as individual mice have highly private tumor TCR repertoires even though they are genetically identical, and were inoculated with ostensibly similar cancer cell lines (60, Nicola Principe et al., 2020) (manuscript under review). This highlights the challenge of developing a TCR based dynamic biomarker of response from tumor samples.

## Nuanced Analysis of PD-1^+^ Blood T Cells Might Offer a Biomarker to Response to PD-1/L1 Blockade

Peripheral blood T cells are heterogeneous and include naïve, effector and memory T cells. Bulk TCR sequencing is often performed on all PBMCs, and TCR distributions within these different T cell subsets are lost. This could explain why some studies show an association between TCR diversity and response to ICT, but not others. Focusing TCR diversity analysis on a phenotypically distinct subset of blood T cells could provide a more accurate biomarker of response, compared to analysis of whole blood samples. Peripheral PD-1^+^ T cells represents one such population, as CD8^+^PD-1^+^ are the primary T cells which PD-1 blockade acts, are clonally expanded, and are enriched for tumor-specific T cells (65–67). TCR diversity of select populations of T cells can be derived from sequencing flow cytometry sorted populations. Patients with high pre-treatment TCR diversity, and reduced diversity post anti-PD-1 treatment in their CD8^+^PD-1^+^ T cell population had longer progression-free survival. Importantly, these associations with treatment outcomes were not observed when TCR sequencing was performed on blood CD8^+^ T cells (66, 68).

## Expansion of Intra-Tumoral TCR Clonotypes is a Feature of Response to Anti-PD-1 Therapy

A pre-treatment tumor TCR*β* repertoire with reduced diversity correlates with clinical response to anti-PD-1 therapy in some melanoma, and lung cancer patient cohorts (13, 17, 40), but not others (69, 70). In pancreatic ductal carcinomas, post-treatment but not pre-treatment TCR*β* clonality was associated with response to anti-PD-1 (43). Patients who were refractory to anti-CTLA-4 therapy, but who responded to subsequent anti-PD-1 therapy had a more clonal TCR*β* repertoire before and after PD-1 blockade (57). In studies where subsequent biopsies could be profiled, expansion of a greater number of TCR*β* clonotypes between pre- and on-treatment samples was observed in responders compared to a smaller number of expanded clonotypes in non-responders (13, 40, 57, 70). Even though the timing of biopsies varied between studies, expansion in tumor TCR*β* clonotypes suggests antigen-specific T cell proliferation and is likely to be a key feature of successful responses to anti-PD-1 therapy.

Some studies have described dynamic changes in tumor and blood TCR repertoire, providing insight into how the T cell response changes when ICT treatment is administered. However, the utility of the TCR repertoire as a dynamic biomarker of ICT response is still limited, and we discuss some of the current limitations in the next section.

## Improving How TCR Repertoire Data is Represented

**Figure 2:**
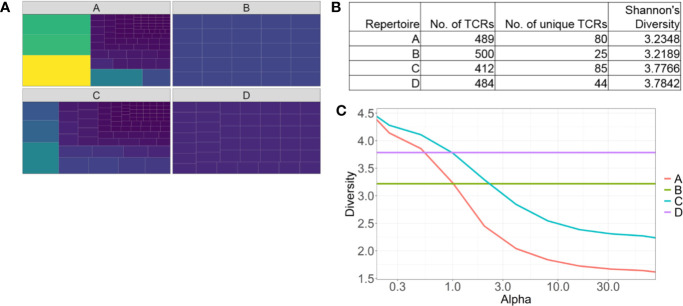
Identifying pitfalls in one-dimensional T cell receptor diversity metrics. **(A)** Tree maps of four examples of TCR repertoires. Each tile is a unique clone, and the size of each tile represents the abundance of each clone. **(B)** Table of summary statistics of the four repertoires, highlighting different distributions of TCRs can have similar diversity scores. **(C)** Representative Renyi diversity plots of the four repertoire examples. Features of each line/curve on the plot, such as the gradient and where the curve intersects the y-axis, correspond to different repertoire features.

The most frequently used diversity metrics, including Shannon’s and Simpson’s indices are useful to generate a single numerical score to estimate repertoire diversity of millions of TCR sequences (71). However descriptive information, such as oligo- or monoclonality is lost when data is compressed like this. For example, two repertoires with the same numerical Shannon’s or Simpson’s diversity values can have vastly different repertoires, especially in how the most abundant clones are distributed (**Figures 2A, B**). Renyi entropy can be used to graphically represent how abundant clones are distributed in relation to the rare clones within a given repertoire, in addition to assigning a numerical value to repertoire diversity (**Figure 2C**) (72). For example, the gradient of the slope increases as the distribution of the repertoire becomes more monoclonal.

Another common feature of bulk TCR sequencing data is the large number of unique TCR clones that occur once only. These low-frequency TCRs can skew Shannon’s and Simpson’s indices, increasing the diversity score in the presence of a few dominant clones. Ecological diversity metrics were designed to account for rare species, but it is unclear if a rare, unique TCR*β* sequence belongs to a rare T cell clone, or a sequencing artefact. Even if it was the former, it is likely that only the most abundant clonotypes are of biological relevance. Hence, modified diversity metrics that only account for the more abundant TCR clonotypes have been used to characterize repertoires, and stratify patients. Top 10 clones or the top 50% of the most abundant clones have been used to represent TCR repertoire diversity (62). A recent melanoma study highlighted ICT response correlated with a higher number of large clones (clones occupying greater than 0.5% of the total repertoire) in the blood (31).

Diversity indices are highly sensitive to the sequencing depth and absolute number of TCRs sampled in each repertoire. Comparisons of diversity measurements are only meaningful if repertoires have sufficient sampling and similar repertoire sizes. The absolute number of TCRs and unique TCRs from individual samples are important data that should be presented for understanding and comparing TCR repertoire diversity. A challenge with developing a predictive biomarker based on TCR diversity is the variation in how repertoire diversity is measured between different studies and the lack of validation of these metrics used to stratify ICT responders and non-responders.

## Moving Beyond Diversity: Novel Approaches to Study TCR Repertoires

Recent studies have approached TCR repertoire analysis utilizing novel computational approaches. Some unique TCR*β* CDR3 sequences only differ from others by a single or few amino acids. A novel approach to studying TCR repertoires is to account for sequence similarity, with the underlying assumption that TCRs specific for the same antigen will have similar CDR3 amino acid sequences. Clustering highly similar sequences, and mapping them as networks can reveal interesting properties about TCR repertoire structure. The most abundant, public TCR*β* CDR3 sequences are highly conserved between healthy individuals, more so than previously expected (73). When clustering approaches are applied to longitudinal blood samples, changes in the connectivity of TCR clusters over time distinguished healthy and tumor bearing mice (74). ICT possibly alters TCR networks in patients over time (73). Mapping TCR networks based on amino acid motifs within the CDR3 region potentially offers a more robust biomarker of response to therapy than TCR diversity measurement by Shannon’s alone (75).

Some key papers describe how a clustering approach identified shared amino acid motifs within CDR3 regions of common virus (influenza, CMV) epitope specific TCR repertoires (76, 77). If tumor-epitope associated TCR clusters can be identified, the expansion and contraction of these clusters can be tracked over time and might be expected to correlate better with response. Heterogeneity in tumor antigens between patients and the lack of TCR sequencing data of tumor-specific T cells are significant hurdles to this. It is also likely that such an approach would be suited to shared tumor antigens, such as tumor differentiation or viral associated tumor antigens.

As a further consideration, it is important to recognize that the nucleotide sequence within TCR*β* CDR3 regions can be redundant, with receptors having identical amino acid sequences but different nucleotide sequences. TCR convergence in peripheral blood, defined as the frequency of unique TCR*β* nucleotide sequences that share a CDR3 amino acid sequence with at least one other clone, correlated with response to anti-CTLA-4 in a small cohort of patients (42).

High dimensional analysis comparing amino acid residues or nucleotide bases between millions of TCR sequences is now possible, giving us unprecedented information about TCR repertoires and how they relate to anti-tumor immunity. Although these approaches are at an early stage, the use of novel analytic approaches could inform our understanding of the TCR repertoire structure in the context of tumor immunology and assist development of a TCR based dynamic biomarker of response.

## Clonal Diversity of Memory CD8^+^ T Cell Subsets Might Offer a Dynamic Biomarker of ICT Response

Another approach to develop a novel biomarker of response is to focus on the clonal diversity within a population of relevant T cells. Most existing studies perform bulk TCR sequencing on whole blood and tumor samples, without the capacity to differentiate heterogeneous T cell populations that differ in phenotype and function. We would anticipate that changes in TCR diversity, such as clonal expansion within a population of T cells would be more robust biomarkers of response to ICT, compared to TCR diversity derived from unsorted T cells.

The advent of single-cell technology that incorporates transcriptome and TCR profiling now permits the assignment of T cell clones to a phenotype or phenotype cluster. This has the potential to provide unprecedented insight into the dynamics of phenotype change within T cell clones and how those changes relate to ICT treatment response. Recent papers highlight the role of memory CD8^+^ T cells in ICT therapy, identifying tissue resident memory (TRM) and effector memory-like CD8^+^ T cell subsets as potential predictors of positive outcomes to ICT therapy.

A specialized subset of resident memory T cells reside in tissues to mount an effective, rapid local immune response. TRM cells are canonically identified by surface expression of CD69 and CD103 and reside in organs such as the skin and lung and within solid tumors. CD8^+^CD103^+^ tumor infiltrating T cells often correlate with better prognosis and outcomes in multiple cancers (68, 78–87). Clonally expanded TRM expressed PD-1 and correlated with effective anti-PD-1 therapy in lung cancer patients. Single-cell analysis of TRM from these responding patients demonstrated increased expression of cytotoxicity genes in these populations (88).

Single-cell analysis of CD8^+^ T cells from melanoma biopsies revealed that ICT responders are enriched with effector memory like T cells that express genes associated with memory, activation and cell survival (IL-7R, TCF7), but not residency (89). The transcription factor TCF7 is critical for CD8^+^ T cell proliferation, differentiation (90), especially in the context of anti-PD-1 therapy (91, 92). Importantly, TCF7 expression was validated in a different patient cohort and found to associate with responses to ICT (89). A limitation to acquiring dynamic data of TRM or TIL subsets is the difficulty in obtaining serial on-treatment tumor biopsies for study.

When serial peripheral blood T cells were analyzed using single-cell phenotyping, responders to both anti-PD-1 and anti-PD-1/anti-CTLA-4 exhibited more expanded T cell clones than non-responders in PBMCs post-treatment (31). Some highly expanded clones appeared to differentially express genes associated with cytotoxicity (such as granzyme B, perforin, ITGB1, and CCL4), compared to the non-expanded clones (31). In another study, a subset of clonally expanded memory T cells characterized by CD27^−^CCR7^−^ were found in the peripheral blood of patients responding to ICT (93). In these patients, response to immunotherapy correlated with a subset of clonally expanded T cells identified by single-cell RNAseq, representing a potential biomarker of response.

Although single-cell sequencing is a powerful tool, the costs are very high. It does, however, provide novel insights that can be tested with bulk TCRseq and flow cytometry analysis. A more cost effective T cell based biomarker might lie in TCR analysis of a subset of effector T cells. Further work is required to identify surface markers that define memory T cell subsets of interest across multiple studies. Lastly, the approach of combining TCR sequencing and flow cytometry analysis to develop a biomarker of response is agnostic to antigens and would be useful when tumor antigens are not well defined.

## Conclusion

Immune checkpoint therapy has changed the therapeutic landscape allowing the word “cure” to enter the oncologist’s lexicon at least for a subset of hitherto incurable cancer patients. Methods to stratify cancer patients who will benefit most from therapy are desperately needed because of the high cost of treatment and the potential for adverse reaction without clinical benefit. The specificity of the immune system allows us to leverage T cell receptor sequencing as a potential biomarker of patient outcomes. T cell proliferation and differentiation is a dynamic process so serial samples are likely to boost the predictive potential of TCRseq on ICT outcomes. Sequencing of specific subsets of T cells will also improve predictive power as the downstream effectors and additional targets of ICT are identified. Immune cell phenotyping and receptor sequencing combined in single-cell RNAseq is beginning to provide the greatest depth of analysis and is likely to better stratify patients by predicted outcomes. The current challenge is to make single-cell RNAseq more accessible by overcoming technical barriers and developing robust mathematical and statistical models to accurately interpret the wealth of information. The last 10 years of cancer immunology research have demonstrated an acceleration in treatment, diagnostic and predictive capacity that ensure the future contains better outcomes for all cancer patients.

## Author Contributions

JK wrote the article. JC edited and conceived the article. NP, TL, AN, MW, RH, WL, and RL provided critical review and edited the article. All authors contributed to the article and approved the submitted version.

## Funding

JK receives a stipend from the iCare Dust Diseases Board. JC receives funding from the iCare Dust Diseases Board, Cancer Council Western Australia and the University of Western Australia Raine Foundation. WL is supported by a Simon Lee Fellowship, an NHMRC Fellowship and a Cancer Council WA fellowship. The National Centre for Asbestos Related Diseases receives funding as an NHMRC Centre of Research Excellence.

## Conflict of Interest

The authors declare that the research was conducted in the absence of any commercial or financial relationships that could be construed as a potential conflict of interest.
